# Lethal nitrous oxide (N_2_O) intoxication during surgery: the contribution of immunohistochemistry in identifying the cause of death: a case report

**DOI:** 10.1186/s13256-023-04159-7

**Published:** 2023-10-10

**Authors:** Andrea Cioffi, Camilla Cecannecchia, Maria Antonella Bosco, Giovanni Gurgoglione, Benedetta Baldari, Stefania De Simone

**Affiliations:** 1 Section of Forensic Science, Section of Legal Medicine, Department of Clinical and Experimental Medicine, University of Foggia, Viale Europa 12, 71122 Foggia, Italy; 2 Department of Anatomical, Histological, Forensic and Orthopaedic Sciences, Sapienza University of Rome, Rome, Italy

**Keywords:** Nitrous oxide, N_2_O, Forensic histopathology, Asphyxia, Hypoxia, Selectins, HIF 1-α, Anaesthesia

## Abstract

**Background:**

Nitrous oxide (N_2_O) is a gas used in medicine for its analgesic, anxiolytic and amnesic properties. It is a drug considered safe if adequately administered. In the literature, accidental N_2_O-related deaths are rare. They are mostly related to inhalation of this substance for recreational and autoerotic purposes; rarely are reported deaths due to incorrect administration of medical gas in anesthesia. The diagnosis of death from acute N_2_O intoxication is complex and is generally an exclusion diagnosis: the macroscopic and microscopic post-mortem signs are entirely nonspecific. Furthermore, the circumstantial data are not always supportive and can even be confusing, mainly if the death occurred inside a hospital.

**Case presentation:**

We describe a particular case of death from acute nitrous oxide poisoning in a hospital environment, of a Caucasian male of 72-years-old. The intoxication occurred during a minimally invasive vascular surgery due to an incorrect assembly of the supply lines of medical gases (O_2_ and N_2_O). The identification of the cause of death resulted from the analysis of circumstantial data, macroscopic and microscopic autoptic findings, and immunohistochemical investigations based on the search for antibodies anti E-selectin, P-selectin, and HIF 1-α.

**Conclusion:**

Although not pathognomonic of asphyxiation by N_2_O, the latter molecules are a valid and early marker of hypoxic insult. Therefore, in concert with all other findings, it may constitute valid support for the forensic pathologist to ascertain the cause of death in case of suspected intoxication by N_2_O.

## Introduction

Nitrous oxide (N_2_O) is a colourless, tasteless, and odourless gas, highly fat soluble, chemically inert, and bacteriostatic, used as an inhaled anaesthetic in surgery and dentistry since 1800 [1].

N_2_O is also known as "laughing gas" for its euphoric, joyful, and sometimes hallucinogenic effects; these are associated with an alarming and increasing spread of gas as a substance of abuse [2, 3]. Generally, for recreational purposes, the inhalation occurs through bottles or N_2_O-filled balloons, called funky balls [4].

As a medical gas with analgesic, anxiolytic and amnesic properties, it is used for conscious sedation in dentistry, minor surgeries, burns, and during labour. In addition, in combination with other analgesics, it is used for general anaesthesia [5–7]

The action mechanism of N_2_O is not yet fully understood; however, the most credited theory is that the gas produces anaesthetic effects by uncompetitive inhibition of the *N*-methyl d-aspartate (NMDA) receptors. Thus, for the analgesic action, the gas inhibits glutamatergic excitatory neurotransmission [7–9].

N_2_O is not very soluble, so it is absorbed and eliminated by the lungs. This property explains the short latency of the analgesic effect and the rapid return of the initial mental state of the patient. Nitrous oxide has no metabolites, so the sedative effect is rapid [10].

The clinical effect of N_2_O depends on the administered concentration. If its concentration is less than 50%, it produces an analgesic, sedative, and anxiolytic effect without altering the state of consciousness; instead, anaesthetic concentrations are > 50%.

As anticipated, nitrous oxide cannot induce surgical anaesthesia alone and requires association with other drugs. In fact, despite the Minimal Alveolar Concentration (MAC) of N_2_O being 105%, the need to simultaneously ensure an oxygen supply prevents the use of gas at concentrations > 70%. [11]. High concentrations result in the diffusion of N_2_O out of the blood into the alveoli, causing hypoxemia, coma, and death [12].

Therefore, the safe administration of N_2_O to patients is based on the administration of gas mixtures containing a fair and controlled percentage of oxygen.

Although N_2_O can now be considered a safe anaesthetic drug, its use remains controversial due to its adverse effects. There are several randomized clinical trials to evaluate the perioperative risk associated with the use of N_2_O in general anaesthesia. These studies demonstrated that N_2_O, compared with other agents, is associated with an increased risk of postoperative nausea and vomiting (PONV); however, studies did not demonstrate any significant correlation with perioperative surgical site infectious or cardiovascular complications and increased mortality [13, 14].

There is scientific evidence of toxic effects (neurological and psychiatric) in chronic inhalation due to the inactivation of nitrous oxide of methylcobalamin (vitamin B12) [15].

Few deaths due to inhalation of nitrous oxide are described in the literature. Accidental deaths are primarily due to recreational inhalation of the substance [12, 16–18] and autoerotic activity [19, 20]; death can occur from sudden cardiac arrhythmia or, most commonly, acute asphyxiation due to hypoxia [21].

A relatively limited number of deaths NO_2_-related is attributable with certainty to incorrect administration of medical gas in anaesthesia [22, 23].

In our study, we report a rare case of a patient who died after accidental N2O intoxication during the positioning surgery of vascular endoprosthesis. The patient died after some hours of intensive care. Considering such survival period promoting for their expression, we tested some immuno-histochemical markers of hypoxia to verify if they could constitute a valid support for forensic pathologists to address a diagnosis of death by intoxication by N_2_O.

## Case report

### Circumstantial evidence and clinical history

A 72-year-old Caucasian male patient was admitted to a hospital to undergo a minimally invasive endovascular surgery of bisiliac endoprosthesis implantation because of aneurysmal dilations.

In the past, the patient had already undergone traditional endoprosthetic implantation in the abdominal aorta. Except for this, the patient's medical history was silent for cardiovascular and respiratory diseases. The patient carried out regular physical activity, and his general condition was good. Preoperative anesthesia tests (blood tests, echocardiography, ECG) did not show contraindications to local and general anesthesia. The doctors selected a minimally invasive surgery under local anesthesia because of the probable evidence of abdominal adhesions attributable to previous surgery.

The surgery was performed in a renewed angiographic room; the procedure was carried out in conscious sedation by infiltrating local anesthetics (Naropin and Lidocaine). However, after about 15 min from the surgical incision, the patient, supported by an oxygen mask, was particularly agitated and faced a sudden loss of consciousness with sudden oxygen desaturation (up to 35% for a few minutes), such as required orotracheal intubation. At the same time, there was a reduction in blood pressure and a slight bradycardia, although there was an ECG track in the norm. Subsequently, cardiac arrest was realized on an arrhythmic basis and, consequently, cardiogenic shock (ejection fraction of 15%), which required hemodynamic stabilization by ECMO (Extra Corporeal Membrane Oxygenation).

During the following hours, the patient was transferred to the intensive care unit; the state of consciousness and breathing autonomy were not recovered, and numerous ventricular extrasystoles arose. The death occurred ~ 17 h after surgery.

During the following days, a technical evaluation was carried out on the tubes supplying medical gases at the new angiographic room where the patient had been operated. The assessment indicated the wrong inversion between the compressed air and nitrous oxide circuits. The gas mixture flow delivered to the patient was not administered in the percentages set on the mixer but consisted of 100% nitrous oxide.

### Autoptic findings

The external examination did not show traumatic signs. Internal examination was also carried out.

There were signs of atherosclerosis of the Willis circle in the absence of hemodynamically significant plaques. The brain, weighing 1480 g, was widely edematous.

The whole heart (weighing 420 g) was fixed in a formaldehyde solution. There was the common trunk of the left coronary ectasia with calcific thickening of the vascular wall in the absence of complicated or occluding atheromatic plaques. The opening of the main coronary arteries showed only slight ectasia without occlusion or sub-occlusion of the vascular lumen.

The heart presented dilation of both ventricular chambers with concentric hypertrophy of the left ventricle.

The lungs were greatly expanded, edematous, and slightly congested. The bronco-vascular structures were devoid of pathological elements.

The remaining abdominal organs had no visible changes but showed widespread congestion.

The abdominal aorta had a properly positioned endovascular prosthesis. In correspondence with the left common iliac artery, there was further aneurysm dilation of about 6 cm in diameter.

### Histological findings

Hematoxylin eosin staining was used. The brain showed diffuse interstitial edema.

The myocardium showed moderate neutrophil infiltration, without signs of necrosis and focal areas with contraction bands; vascular structures were congested with the focal presence of peri-vasal hemorrhagic spreads.

Histological investigation of both lungs showed emphysema, edema, and moderate congestion; focal intra-alveolar hemorrhages were also observed.

Histological examination of the other organs did not show signs useful for the identification of the cause of death.

### Immunohistochemical findings

For the IHC, samples were de-waxed, hydrated, and subjected to heat-induced epitope retrieval (HIER) using Target Retrieval Solution (Dako) (20′) in a thermostatic bath at 98 °C. The staining procedure was performed using a Dako Autostainer Instrument, as follows [24, 25]: incubation in peroxide solution (10′); incubation at room temperature (RT) with primary antibody, for P-selectin and E-selectin (30′) in Dako REAL™ EnVision™ Detection System, Peroxidase/DAB + , Rabbit/Mouse; incubation with Dako DAB-AWAY detection system (5′); washed in distilled water; incubation for 1' with Harris hematoxylin. The following molecules were investigated by immunohistochemistry: E-selectin (AB E-Selectin/CD62E/SELE (D-7): sc-137054 of Santa Cruz Biotechnology, inc.), P-selectin (AB P-Selectin/CD62P/SELP (CTB201): sc-8419 of Santa Cruz Biotechnology, inc.), and HIF 1-α (HIF-1 α AB: sc-53546 of Santa Cruz Biotechnology, inc.).

The characteristics of the antibodies are summarized in Table 1.

**Table 1:** Characteristics of the antibodies [45]

E-Selectin/CD62E/SELE (D-7): sc-137054	E-Selectin Antibody (D-7) is an IgG_2a_ κ mouse monoclonal E-Selectin antibody (also designated Selectin E antibody, or CD62E antibody) that detects the E-Selectin protein of mouse, rat and human origin by WB, IP, IF, IHC(P) and ELISA. Selectins, also designated CD62 antigens, comprise a family of carbohydrate-binding proteins involved in mediating cellular interactions with leukocytes. E-Selectin (also designated ELMA-1 or CD62E) is expressed on endothelial cells, exhibit overlapping ligand specificities. E-Selectin is expressed by cytokine-stimulated endothelial cells and is thought to be responsible for the accumulation of blood leukocytes at sites of inflammation by mediating the adhesion of cells to the vascular lining
P-Selectin/CD62P/SELP (CTB201): sc-8419	P-Selectin Antibody (CTB201) is an IgG_1_ κ mouse monoclonal P-Selectin antibody (also designated CD62 antibody, SELP antibody, selectin P antibody) that detects the P-Selectin protein of mouse, rat and human origin by WB, IP, IF, IHC(P) and FCM. Selectins, also designated CD62 antigens, comprise a family of carbohydrate-binding proteins involved in mediating cellular interactions with leukocytes. P-Selectin (also designated GMP-140 or CD62P), expressed on activated platelets and endothelial cells. It is also recognize sialyl-Le (x) as a ligand and bind to specific carbohydrates on neutrophils and monocytes
HIF-1 α AB: sc-53546	HIF-1α Antibody (H1α 67) is an IgG2b κ mouse monoclonal HIF-1α antibody that detects HIF-1α of mouse, rat and human origin by WB, IP, IF and IHC(P). Cell growth and viability is compromised by oxygen deprivation (hypoxia). Hypoxia-inducible factors (including HIF-1α) induce glycolysis, erythropoiesis and angiogenesis in order to restore oxygen homeostasis. In response to hypoxia, HIF-1α is upregulated and forms a heterodimer with Arnt 1 to form the HIF-1 complex

CD: Cluster of differentiation, ELAM 1: endothelial-leukocyte adhesion molecule 1, IgG: Immunoglobulin G, WB: Western Blot, IF: Immunofluorescence, IP: Immunoprecipitation, IHC: Immunohistochemistry, ELISA: enzyme-linked immunosorbent assay, SELP: Selectin P, FCM: flow cytometry, ARNT: Aryl Hydrocarbon Receptor Nuclear Translocator, HIF 1- α: Hypoxia-inducible factor

**Fig. 1:**
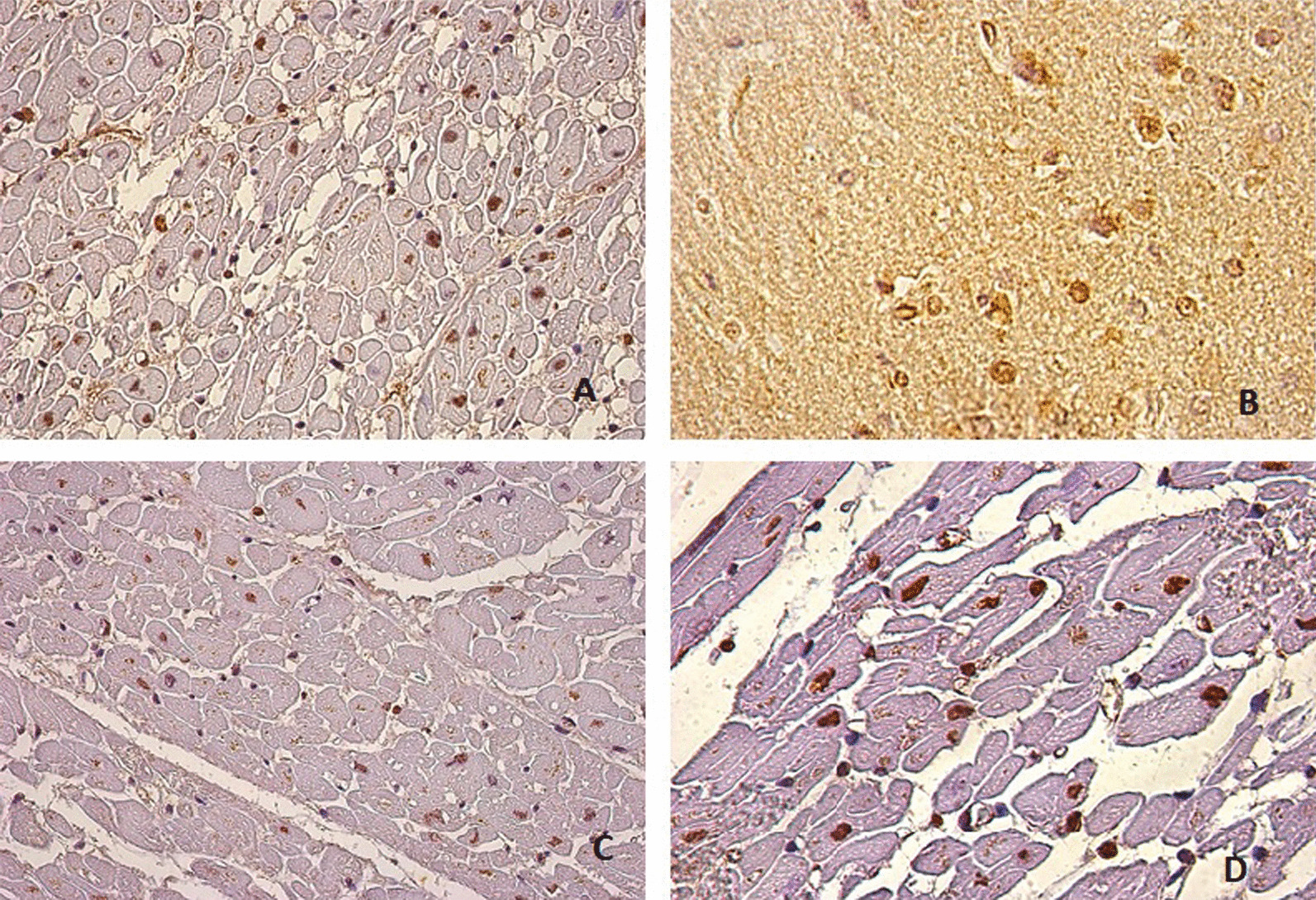
HIF 1-alpha immunohistochemistry nuclear positive reaction. **A**: heart 40x, **B**: brain 63x, **C**: heart 40x, **D**: heart 63x

The heart expressed HIF-1α in the myocardial nucleus areas showing signs of reperfusion, such as the brain (Fig. 1). Areas close to hemorrhagic extravasation and areas with fibrotic replacement also showed HIF-1α.

**Fig. 2:**
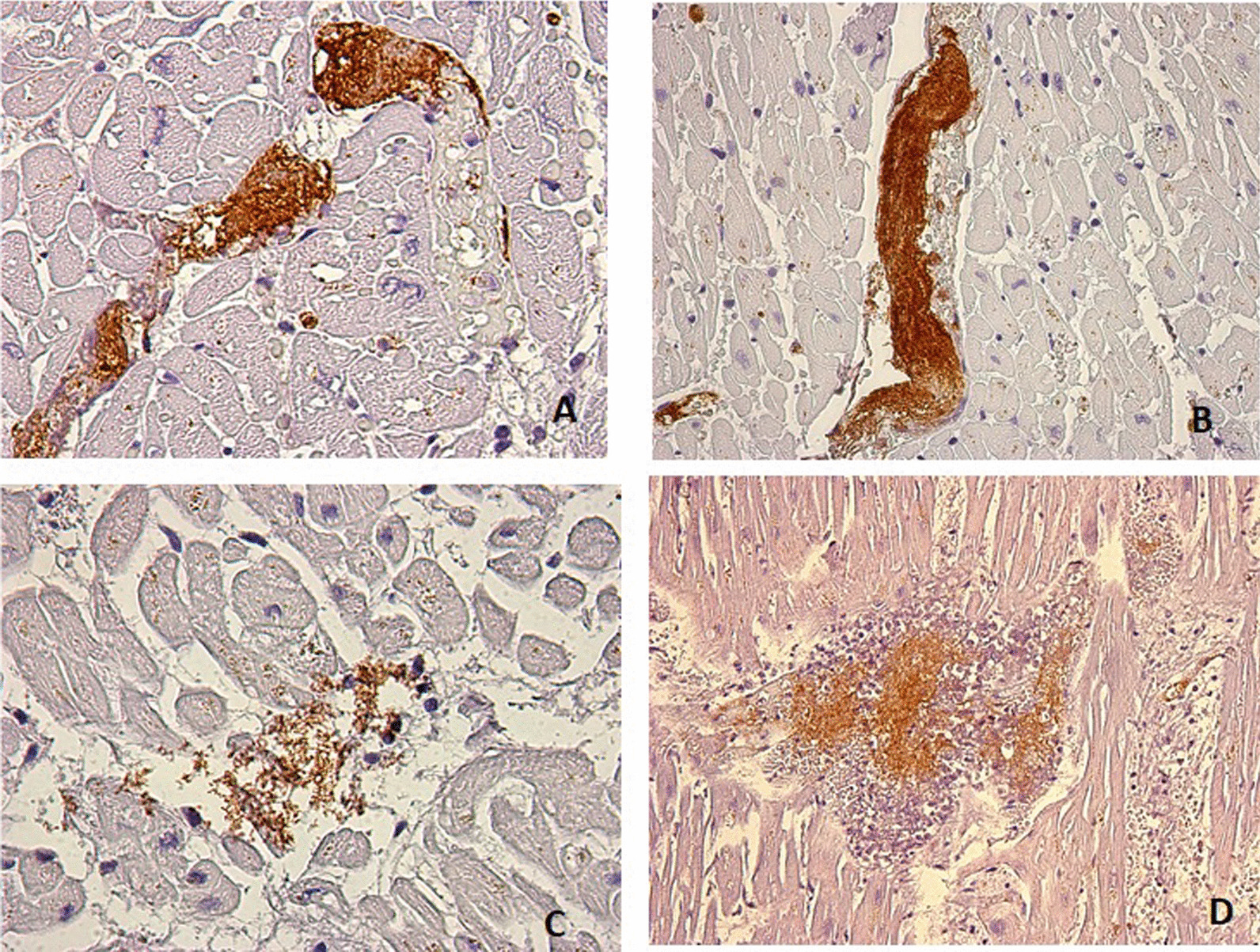
P-Selectin: heart samples strong positive reaction of aggregate platelets, expressed widely in the areas of hemorrhagic extravasation, **A** 63x and **B** 40X. E-Selectin: moderate positive reaction, **C** 63x and **D** 40x

The P-selectin revealed the positivity of aggregate platelets on the heart, expressed widely in hemorrhagic extravasation (Fig. 2A, B). Also, E-Selectin was present in the same fields but less intensely (Fig. 2B, C).

P-Selectin was expressed widely in medium vessels of the emphysematous lungs. Hemorrhagic areas were also positive for Selectin P. That evidence suggests that myocardial tissue has suffered from a lack of oxygen perfusion; the diffuse interstitial infiltration and the poor coloration of the nuclei were compatible with defective cardiac oxygenation. The brain suffered the same hypoxic damage, showing positive for each marker.

According to the literature study [25, 26] the experimental evidence highlights ischemia–reperfusion injury indicative of hypoxia.

Based on circumstantial data, the technical report performed on the tubes of the angiographic room, the autopsy findings and immunohistochemistry, the cause of death was attributed to acute cardiorespiratory failure from accidental intoxication by nitrous oxide.

## Discussion

The reported case regards a fatal nitrous oxide (N_2_O) intoxication that occurred in a patient during surgery under local anesthesia due to an incorrect exchange of medical gas supply lines. This led to the patient being given 100% N_2_O instead of an O_2_ gas mixture during surgery.

Due to the suffocating dose of N_2_O mistakenly delivered through the mask, the patient suffered a loss of consciousness and severe and repeated episodes of desaturation and respiratory failure. Then followed the coma and cardiac arrest on an arrhythmic basis a few hours after surgery.

As anticipated, N_2_O, to be administered safely as an analgesic, must be mixed with an adequate percentage of O_2_ and must not exceed 70%; higher doses are quickly asphyxiating [12]. Nitrous oxide at high concentrations inhibits the physiological response to hypoxia due to oxygen depletion [27].

In the scientific literature, few deaths are attributed with certainty to intoxication by nitrous oxide.

In the hospital environment, deaths from nitrous oxide poisoning are even rarer, and those few, in general, are attributable to malfunction of medical gas delivery machinery or their incorrect use. Few cases of nitrous oxide poisoning due to an erroneous swapping of the lines in the hospital gas system are described in the scientific literature [23].

Poli *et al.* described eight cases of lethal intoxication by N_2_O during the administration of O_2_ at the new Cardiovascular Intensive Care Unit (ICU) of a hospital due to an erroneous swapping of the lines in the gas system (N_2_O instead of O_2_) [22].

The literature also describes a few other hospital deaths related to nitrous oxide; in fact, few deaths occurred in pediatric dental procedures during conscious anesthesia with mixtures of O_2_ and N_2_O [28].

The identification of deaths from acute nitrous oxide poisoning is complex because the macroscopic and microscopic post-mortem signs are rather unspecific and not directly attributable to nitrous oxide poisoning. There are usually nonspecific signs of asphyxia, such as cyanosis, pulmonary emphysema, and cerebral edema, widespread congestion [12, 29, 30].

Therefore, often the autopsy is helpful to exclude the presence of valid causes of death.

Histological examination with hematoxylin and eosin also does not allow the detection of direct and specific signs of nitrous oxide poisoning. It may reveal indirect and subsequent signs of hypoxic organ suffering, such as acute cerebral edema, anoxemic myocardial lesions, and myocardial contraction bands [17, 29]. However, there are few articles in the literature describing lethal N_2_O poisoning. Cipolloni *et al.* [31] resumed the evidence finded in cases of N_2_O intoxication, reported in Table 2.

**Table 2:** Summarized of the evidence finded in cases of N_2_O intoxication

Unknow death	
Crime scene investigation	Presence of N_2_O cylinder on site connected to a tube, or whipped canister, or plastic bag. Anamnesis of recent anasthesia
Autoptic examination	Cyanosis, conjunctival petechiae, visceral congestion, and pulmonary edema. Exclusion of traumatic lesions
Histological examination	Cerebral edema, widespread stasis, contraction bands in the myocardium. Exclusion of hemorrhage or other traumatic evidence
Immunohistochemical examination	Positivity of ischemia–reperfusion markers indicative of hypoxia
Toxicological examination	Finding of N_2_O in blood and urine. Exclusion of other cause of intoxication (alcohol, drugs, etc.)

In our case, the autopsy revealed signs of cerebral edema, edematous and expanded lungs, and polyvisceral congestion; these results are similar to the very few cases already described in the literature. Histological investigations confirmed the presence of widespread cerebral edema and showed signs of congestion and ischemia–reperfusion damage (bands of contraction, infiltration of neutrophil granulocytes, vascular congestion with perivasal hemorrhagic spread); at the pulmonary level, there was widespread edema, emphysema and moderate congestion.

The ischemia–reperfusion damage occurs when restoration of oxygenated blood in an ischemic tissue takes place; in this process, massive recruitment of leukocytes occurs at the site of damage. The selectins and their ligands are essential for leukocyte tethering/rolling on endothelial cells and the initiation of inflammatory response. In particular, E- and P-selectin are predominant in leukocyte recruitment to inflammatory sites [32–34].

Thus, E- and P-selectin are considered markers of the early inflammatory response to hypoxic insult [35], and Tumor necrosis factor α (TNF-α) increases the intracellular adhesion molecule expression [36]. Inhalation anesthetics (such as nitrous oxide) do not affect TNF-α induced E-selectin expression [21]. Moreover, in the case of hypoxic insult, the first response consists of the transcription of the Hypoxia Inducible Factor 1-Alpha (HIF-1 Alpha) [37]; it can be considered an early marker of hypoxia [38, 39]. HIF-1 α is expressed on pulmonary vessels in subjects who died of asphyxia [25], such as E-Selectin in the lungs and brain [40–43].

The antibodies used are not specific for N_2_O intoxication, but can help in the diagnosis, as the ischemia–reperfusion injury is a consequence of the intoxication. For example, E-Selectin and P-Selectin could be positive in any case of inflammation, such as chronic and acute inflammation processes, atherosclerosis or also cancer metastasis [44]. Both selectins and HIF 1-α can be used as indicator of asphyxiated death [45].

To the best of our knowledge, in no case of N_2_O-related death, immunohistochemical markers are used as support to search for the cause of death.

Based on the above-described scientific evidence, we integrated macroscopic, microscopic, and immunohistochemical investigations on lungs, heart and brain using antibodies for E-selectin, P-selectin, and HIF 1-α. In all these organs, such antibodies were expressed; in particular, the brain and heart expressed HIF-α, while P-selectin and E-selectin were more expressed in the lungs and the areas of myocardial hemorrhagic extravasation. According to the literature study [25, 26] the experimental evidence highlights ischemia–reperfusion injury indicative of hypoxia.

Our study demonstrate that immunohistochemical investigations—together with the analysis of the circumstantial data and the results of the autopsy and histology—are helpful to identify the patient's cause of death, in cases suspected to be due to the irreversible consequences of the acute intoxication caused by nitrous oxide.

## Conclusions

Deaths from nitrous oxide poisoning are rare and rarely described in the literature.

The limited number of nitrous oxide-related deaths may be partly explained because they often remain undiagnosed. In fact, in such types of deaths, macroscopic and microscopic post-mortem signs are relatively nonspecific, and often the diagnosis is of exclusion and is formulated based on circumstantial data. Moreover, especially in the case of death in hospitalized patients, the cause of death is further complicated by the presence of confounding factors such as comorbidity, simultaneous use of several drugs, and inherent risk of morbidity and mortality related to surgery. This could lead to an erroneous attribution of the cause of death.

Although there are no specific immunohistochemical markers of N2O asphyxiation, immunohistochemistry may help the forensic pathologist confirm hypoxic damage in case of suspected intoxication. Markers such as HIF-1 α, E-Selectin, and P-Selectin, can be helpful diagnostic tools together with the careful analysis of the detailed data, the subject's history, and the macroscopic and microscopic autopsy findings.

## Data Availability

Not applicable.
